# Long Non-Coding RNA Expression Profile Alteration Induced by Titanium Dioxide Nanoparticles in HepG2 Cells

**DOI:** 10.3390/toxics10120724

**Published:** 2022-11-25

**Authors:** Jiaqi Shi, Yi Zhang, Ying Ma, Zhangjian Chen, Guang Jia

**Affiliations:** 1 Department of Occupational and Environmental Health Sciences, School of Public Health, Peking University, Beijing 100191, China; 2 Beijing Key Laboratory of Toxicological Research and Risk Assessment for Food Safety, School of Public Health, Peking University, Beijing 100191, China

**Keywords:** titanium dioxide nanoparticles, hepatotoxicity, lncRNAs, epigenetics, RNA sequencing

## Abstract

The liver is considered the major target organ affected by oral exposure to titanium dioxide nanoparticles (TiO_2_ NPs), but the mechanism of hepatotoxicity is not fully understood. This study investigated the effect of TiO_2_ NPs on the expression profile of long non-coding RNA (lncRNA) in hepatocytes and tried to understand the potential mechanism of hepatotoxicity through bioinformatics analysis. The human hepatocellular carcinoma cells (HepG2) were treated with TiO_2_ NPs at doses of 0–200 μg/mL for 48 h and then RNA sequencing was implemented. The differential lncRNAs between the control and TiO_2_ NPs-treated groups were screened, then the lncRNA–mRNA network and enrichment pathways were analyzed via multivariate statistics. As a result, 46,759 lncRNAs were identified and 129 differential lncRNAs were screened out. Kyoto Encyclopedia of Genes and Genomes (KEGG) pathway analysis showed that the targeted mRNAs of those differential lncRNAs were enriched in the Hedgehog signaling pathway, Vasopressin-regulated water reabsorption, and Glutamatergic synapse. Moreover, two lncRNA–mRNA networks, including lncRNA NONHSAT256380.1-JRK and lncRNA NONHSAT173563.1-SMIM22, were verified by mRNA detection. This study demonstrated that an alteration in the lncRNA expression profile could be induced by TiO_2_ NPs and epigenetics may play an important role in the mechanism of hepatotoxicity.

## 1. Introduction

Because of favorable mechanical properties and biocompatibility, nanomaterials have been applied to many aspects of our life and work, such as biomedicine [1], the food industry [2], and electronics [3]. The toxicity of nanomaterials has been increasingly studied [4,5]. Titanium dioxide nanoparticles (TiO_2_ NPs) are one of the most widely used nanomaterials. A study detected the titanium particles in a human post mortem liver and spleen and found that more than 24% of TiO_2_ was nanoscale [6]. More and more studies have found that TiO_2_ NPs can be cytotoxic [7,8] and genotoxic [9]. Oxidative stress was induced through the stimulating redox interactions, leading to DNA damage and genomic instability [10,11]. Moreover, there is a growing interest in the in vitro epigenetic changes induced by TiO_2_ NPs [12,13]. TiO_2_ NPs are one of the most commonly used nanomaterials in food additives, pharmaceuticals, and personal hygiene products, such as toothpaste [14], so oral exposure is more likely to happen. The liver is a multicellular organ that plays an important role in activating and eliminating many metabolites; therefore, the liver is the primary target organ of oral exposure to TiO_2_ NPs [15,16,17,18]. Many in vivo experiments found that oral exposure of TiO_2_ NPs can cause liver damage, hepatocyte necrosis, and liver function damage in mice [19,20]. Moreover, some studies concluded that acute toxicity of rats with TiO_2_ NPs induced adverse effects in the liver [21,22]. However, the key mechanism of hepatotoxicity induced by TiO_2_ NPs is not been fully understood and needs further study.

In addition to cytotoxic and genotoxic effects, nanoparticle-induced epigenetic changes and the epigenetic mechanisms behind observed toxicity have also attracted increasing attention. Some studies have found that exposure to nanomaterials can lead to epigenetic changes [23,24]. Pogribna et al. investigated the effect of TiO_2_ NP exposure on histone modifications, a major epigenetic mechanism in human colorectal (Caco-2) and lung (NL20) epithelial cell lines, and found changes in several histone modifications after exposure to TiO_2_ NPs [23]. Epigenetics is an important link between genotype and phenotype and plays a key role in the regulation of numerous cellular processes. The main mechanisms of epigenetics include DNA methylation, histone modification, and non-coding RNAs [25]. Non-coding RNAs (ncRNAs) refer to functional RNA molecules that cannot be translated into proteins, among which common regulatory non-coding RNAs include microRNAs (miRNAs), PIWI-interacting RNAs (piRNAs), and long non-coding RNAs (lncRNAs). Many ncRNAs can regulate gene expression through interactions with other epigenetic processes, such as histone modification, chromatin remodeling, and DNA methylation [26,27].

LncRNAs are non-coding RNAs whose transcript lengths range from 200 nt to 100 kb and are one of the key factors in gene transcriptional regulation, affecting all aspects of cellular homeostasis [28]. LncRNAs affect nearly all fundamental processes in living cells, including chromatin formation, replication, transcription, splicing, translation, and post-translational modification, and constitute the richest part of the transcriptional genome [29]. According to the position of lncRNA in the genome to nearby messenger RNAs (mRNAs), lncRNAs can be divided into the following five types [30]: long intergenic noncoding RNAs (lincRNAs), natural antisense transcripts (NATs), overlapping, bidirectional, and sense intronic. LincRNAs are open chromatin structures with transcripts less than 10 kb in length and do not appear at any protein-coding site [31]. LincRNAs are also the most numerous of the lncRNA types [32]. NATs are lncRNAs that block splice-site recognition and recruit epigenetic modifiers [33]. Overlapping transcripts are transcribed in the same direction as a protein-coding gene and contain one protein-coding gene [32]. Bidirectional transcripts compete for transcription initiation and promote chromatin modification of target genes [34]. Sense intronic transcripts are introns derived from a protein-coding gene and their transcription direction is the same as that of the neighboring protein-coding gene [30]. A significant amount of evidence suggests that lncRNAs regulate gene expression in multiple ways on the levels of epigenetic, chromatin remodeling, transcription, and translation [35] and they are potential biomarkers for diagnosing, prophesizing, and monitoring disease progression [36,37,38]. Because of the lower levels of splicing, polyadenylation, and nuclear localization, it is more complex to detect and quantify lncRNAs [35].

To fully assess the toxicity of TiO_2_ NPs, it is critical to assess the epigenetic role of TiO_2_ NPs. However, there is no report yet to explore the function of lncRNAs in the process of TiO_2_ NP-induced toxicity. Therefore, this study treated HepG2 cells with 100 μg/mL TiO_2_ NPs for 48 h and investigated the changes in lncRNAs.

## 2. Materials and Methods

### 2.1. Characterization of Nanomaterials

The TiO_2_ NPs used in this study were obtained from Shanghai Macklin Biochemical Co., Ltd. (Shanghai, China). The detailed characterization methods and physicochemical properties of TiO_2_ NPs were described in our published paper [39]. JEM–1400 electron microscope (JEOL Company, Tokyo, Japan) was used to measure the equivalent diameter. X-ray powder diffractometry (XRD, PANalytical’s X’Pert PRO, X’Celerator, Almelo, The Netherlands) was used to test the crystal form. Dynamic light scattering instrument Zetasizer Nano ZS90 (Malvern Instruments Ltd., Malvern, UK) was used to measure the hydrated particle size and Zeta potential in the serum-free medium containing 1 mg/mL TiO_2_ NPs.

### 2.2. Cell Culture

Human hepatocellular carcinoma cells (HepG2), obtained from the National Biomedical Experimental Cell Resource Library of China, were routinely cultured in Minimum Essential Medium (MEM, HyClone, Thermo Scientific, Logan, UT, USA) supplemented with 10% fetal bovine serum (FBS, Hyclone, Thermo Scientific, Logan, UT, USA), 1% MEM Non-Essential Amino Acids Solution (100×) (NEAA, Gibco, Thermo Scientific, Logan, UT, USA), and 2% GlutaMAX-1 (Gibco, Thermo Scientific, Logan, UT, USA). For subculturing purposes, the cells were digested by 0.25% trypsin and seeded to 96-well plates at a density of 1 × 10^4^ cells per well or 60 × 15 MM plates with 5 × 10^5^ cells per well.

### 2.3. Cytotoxicity Assay Study

Cell Counting Kit-8 assay (CCK-8, Biotopped, Dojindo Laboratories, Kumamoto, Japan) was used to determine the cytotoxicity of TiO_2_ NPs, based on the measurement of the amount of methotrexate generated proportional to the number of living cells. After exposure to 0, 1.5625, 3.125, 6.25, 12.5, 25, 50, 100, and 200 μg/mL TiO_2_ NPs for 48 h, the cells in the 96-well plate were incubated with CCK-8 solution for 2 h. After collecting the supernatants, a microplate reader was used to detect the value of absorbance at 450 nm, taking 600 nm as a parameter. The computation formula is as follows: cell viability = (E − B)/ (C − B). E refers to the experimental hole (containing cell, culture medium, CCK 8, and different concentrations of TiO_2_ NPs), C refers to the control hole (containing cell, culture medium, and CCK 8), and B refers to a blank hole without any cells and TiO_2_ NPs.

### 2.4. Construction of cDNA Libraries and RNA Sequencing

Every control and treatment group set up three repeat samples and then the samples were collected for RNA extraction. The extracted total RNA was qualified by Agilent 2100 Bioanalyzer (Agilent Technologies, Santa Clara, CA, USA) and purified by RNAClean XP Kit (Cat A63987, Beckman Coulter, Inc., Kraemer Boulevard, Brea, CA, USA) and RNase-Free DNase Set (Cat#79254, QIAGEN, GmBH, Dusseldorf, Germany).

The purified total RNA was carried out with rRNA removal, fragmentation, first-strand cDNA synthesis, second-strand cDNA synthesis, end repair, 3′ end plus A, ligation joint, and enrichment. The cDNA was then sequenced with a high-throughput sequencer (Illumina Hiseq 2000/2500, San Diego, CA, USA).

### 2.5. Identification and Quantification of lncRNAs

Gffcompare (version 0.9.8) was applied to identify new transcripts that did not match known annotations and three types of transcripts were picked out with the conditions that transcription length was greater than or equal to 200 bp, the number of exons was greater than or equal to 2, and open reading frame (ORF) was less than 300 bp. Then, Contrastive Predictive Coding analysis (CPC), Coding-Non-Coding Index (CNCI) analysis, and Pfam protein domain analysis were performed to predict the lncRNAs. CPC used supervised machine learning to establish a classification model by learning peptide chain length, amino acid composition, protein homology, secondary structure, protein alignment, or expression [40]. Its classification model was mainly based on the characteristics of sequence ORF length and protein homology; Pfam was a large database of protein family collections, represented by multiple sequence alignments and hidden Markov models (HMMs) [41]. The assembled transcript sequence was annotated by the PfamScan tool. If the sequence matched the Pfam protein database, it was mRNA, and there was no comparison on lncRNA. CNCI identified coded and non-coding sequences by analyzing adjacent nucleotide triplets [42]. Then the transcript with CPC score < 0 and CNCI score < 0 and insignificant results of Pfam was picked out as potential lncRNAs. Finally, it was merged with the NONCODE data database (version: NONCODE 2016; http://www.noncode.org/, accessed on 15 November 2020) and the known lncRNAs in the Ensembl database to form the lncRNA sequence for subsequent analysis.

String tie (version: 1.3.0) was applied to quantify the expression of lncRNA sequences. Then edgeR was applied for differential lncRNA analysis between samples and the *p*-value was corrected through a multiple-hypothesis test, and the q-value was the corrected *p*-value by controlling FDR (False-Discovery Rate). The differential expression multiple fold change was calculated based on the FPKM value. The differential lncRNA filters were as follows: *q*-value ≤ 0.05 and fold change ≥ 2.

The structure of lncRNA and mRNA was compared and analyzed by comparing the differences in transcript length, exon number, and expression level of lncRNA and mRNA. The difference between lncRNA and mRNA molecules was obtained and the predicted lncRNA molecules were verified.

Trans regulation and Cis regulation were used for target gene prediction. Cis referred to how lncRNA regulated neighboring mRNAs (e.g., on the same chromosome) and trans referred to targets at the distal position of chromosomes after different chromosomes.

Finally, KEGG enrichment (https://www.kegg.jp/, accessed on 15 November 2020) was used to analyze the target gene analysis of the differential lncRNA. The selected differentially expressed genes were mapped to each term of the KEGG database, the number of genes for each entry was calculated, and then a super geometric test was applied to a threshold of *p*-value ≤ 0.05 after correction by multiple-hypothesis tests, and the KEGG term that satisfied this condition was defined as the KEGG term that was significantly enriched in the differentially expressed genes.

### 2.6. Statistical Analysis

The numerical data were presented as mean ± standard deviation (m ± SD) of at least three determinations. The statistical analysis was performed by R 3.1.3. A *p*-value less than 0.05 was defined as statistical significance.

## 3. Results

### 3.1. Identification of TiO_2_ NPs

**Figure 1:**
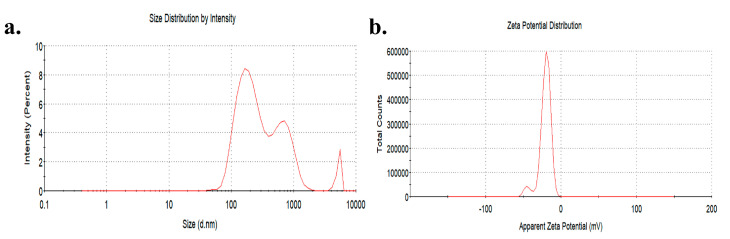
Distribution of hydration particle size (**a**) and zeta potential (**b**) of TiO_2_ NPs in a serum-free medium.

The TiO_2_ NPs used in the study were spherical and anatase type. Transmission electron microscopy (TEM) showed that the equivalent diameter of TiO_2_ NPs was 25.12 ± 5.64 nm. The hydrated particle size of TiO_2_ NPs (1 mg/mL) in a serum-free medium was 323.50 ± 85.44 nm and the zeta potential was −21.00 ± 0.72 mV (Figure 1).

### 3.2. Cytotoxicity of TiO_2_ NPs in HepG2 Cells

**Figure 2:**
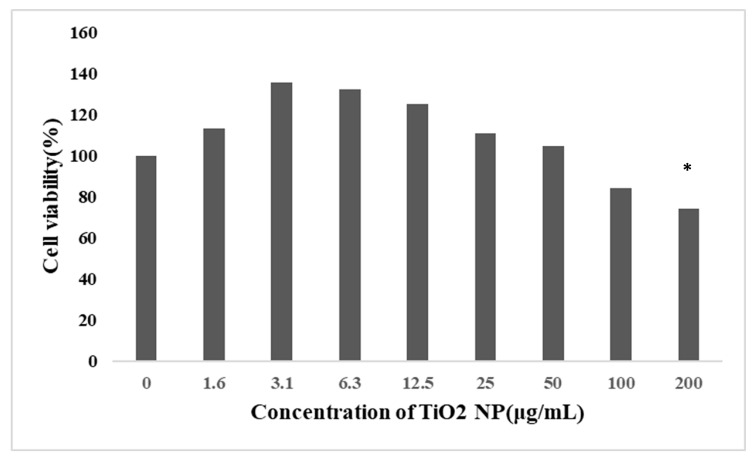
Effect of TiO_2_ NPs on the viability of HepG2 cells (mean ± SD, *n* = 3). Each group set up three repeat biological samples. HepG2 cells were treated with TiO_2_ NPs at 0, 1.5625, 3.125, 6.25, 12.5, 25, 50, 100, and 200 μg/mL for 48 h. The cell viability was significantly decreased in the treatment groups at a concentration of 200 μg/mL. Cell viability did not decrease in a dose-dependent relationship. Significant difference from the control (* *p* < 0.05).

After 48 h exposure, the cell viability decreased gradually with an increase in the concentration of TiO_2_ NPs, and the cell viability of the 200 μg/mL group (65.25%) decreased significantly compared with the control groups, but because the cell viability of the 200 μg/mL group was too low, the final choice was 100 μg/mL (74.16%) as the concentration of the TiO_2_ NP treatment groups (Figure 2). 

### 3.3. Predictions and Annotations of lncRNA-Seq Data

**Figure 3:**
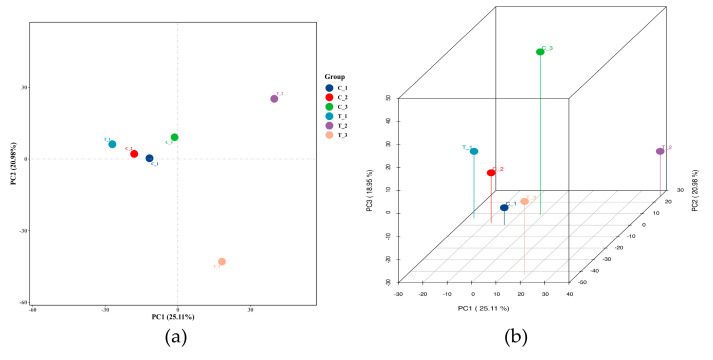
lncRNA expression analysis of TiO_2_ NPs with a concentration of 0 and 100 μg/mL. Princi-pal component analysis (PCA) 2D (**a**) and 3D (**b**) plots were drawn based on the expression of trusted lncRNAs to compare the difference between the control and the treatment groups.

A total of 46,759 lncRNAs, including known and predicted lncRNAs, was identified. According to the position relationship of lncRNAs in the genome to nearby mRNAs, the number of intronic_sense, intronic_antisense, exonic_sense exonic_antisense, intergenic, and bidirectional RNA was 5089, 1621, 12,782, 9855, 12,829, and 4583, respectively. Further, 27.4% of the lncRNAs were lincRNAs, ranking first. Principal component analysis (PCA) scoring plots revealed that the control group and the treatment groups were separated, which represented the difference in lncRNA characteristics (Figure 3).

**Figure 4:**
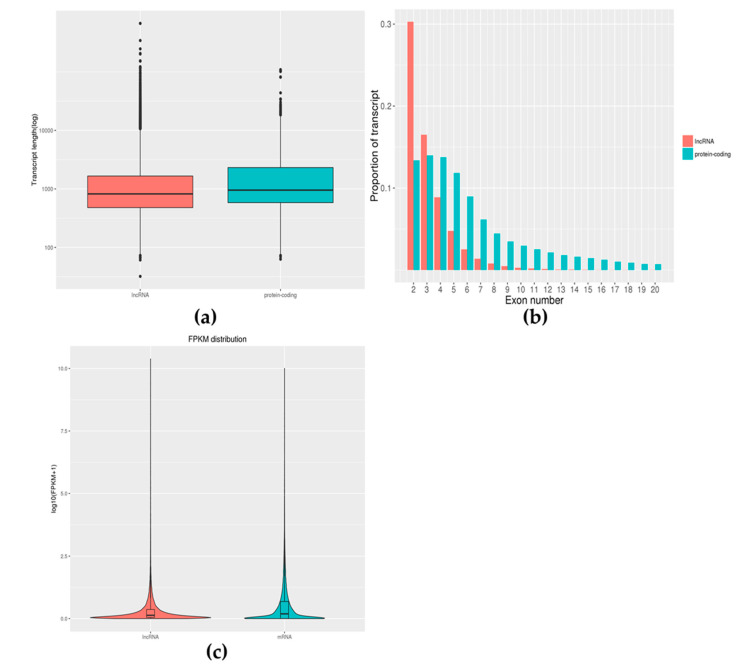
Feature comparison of lncRNA with mRNA. Comparative analysis of the length distribution of lncRNA and mRNA was performed and we found that lncRNA was slightly shorter than the mRNA (**a**). Comparative analysis of exon numbers of lncRNA and mRNA was performed and we found that mRNAs contained a larger range of exons (**b**). The expression values of lncRNAs and mRNAs were averaged separately and the box pattern was plotted with the values of both log10 (FPKM + 1) (**c**). Comparing the expression levels of lncRNAs and mRNAs, it was found that there was a difference in the expression levels of the two.

The length of the lncRNAs was between 32 and 674,512 bp and the median length was 821 bp. As shown in Figure 4a, lncRNA was slightly shorter than the mRNA (median length is 953 bp). Approximately 30.3% of lncRNAs contained two exons, while mRNAs contained several exons from 1 to 363 (Figure 4b). Expression-level analysis showed that the overall expression level of lncRNA was slightly lower than the expression level of mRNA (mean 0.31:0.52, Figure 4c).

### 3.4. Analysis of Differential Expression of lncRNA

**Figure 5:**
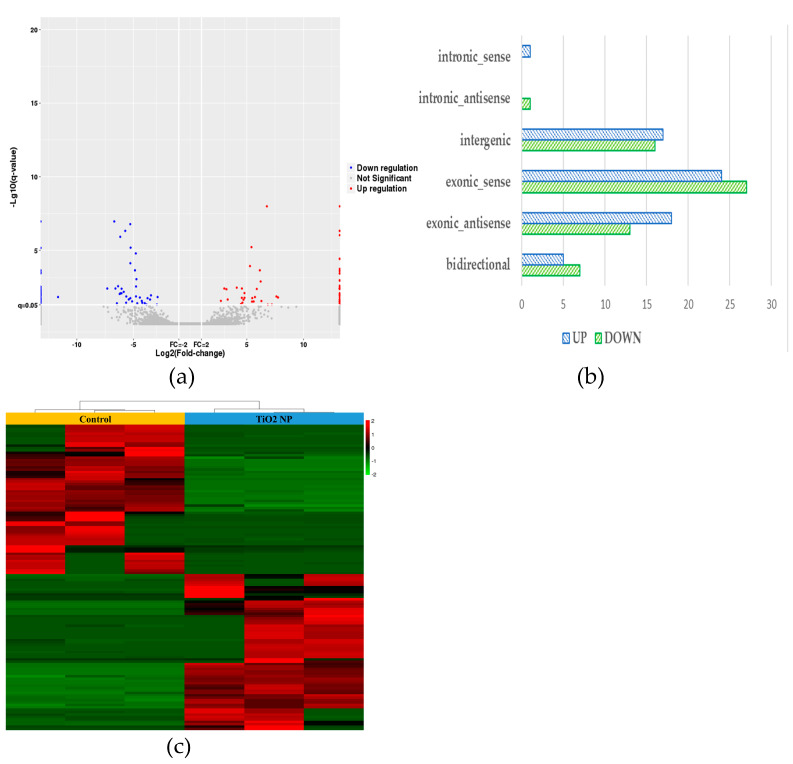
lncRNA differential expression analysis of TiO_2_ NPs with concentrations of 0 and 100 μg/mL. A volcanic map of differentially expressed genes in the treatment group showed the number of up-regulated and down-regulated genes (**a**). The histogram of the relative expression of differential lncRNAs was drawn and showed that the lncRNAs belonging to the exonic-sense class counts had the highest proportion (**b**). A heat map of cluster analysis between the treatment group and control group demonstrated their characteristic difference (**c**).

Finally, 129 differential lncRNAs were screened, of which 65 were up-regulated and 64 were down-regulated (Figure 5a). Among the differential lncRNAs, there was 1 belonging to intronic sense,1 intronic antisense, 51 exonic sense, 31 exonic antisense, 33 intergenic, and 12 bidirectional, respectively (Figure 5b). The cluster heat maps of the differential lncRNAs in the TiO_2_ NP treatment groups compared with the control group are shown in Figure 5c, suggesting that the effect on lncRNA was different between the treatment and control groups. According to the expression profile of lncRNAs in human tissues in the NONCODE database, the up-regulated lncRNAs were mainly expressed in the testes and placenta and the down-regulated lncRNAs were mainly expressed in the adrenal, kidney, and brain.

### 3.5. Enrichment Analysis of Differential lncRNA Target Genes

**Figure 6:**
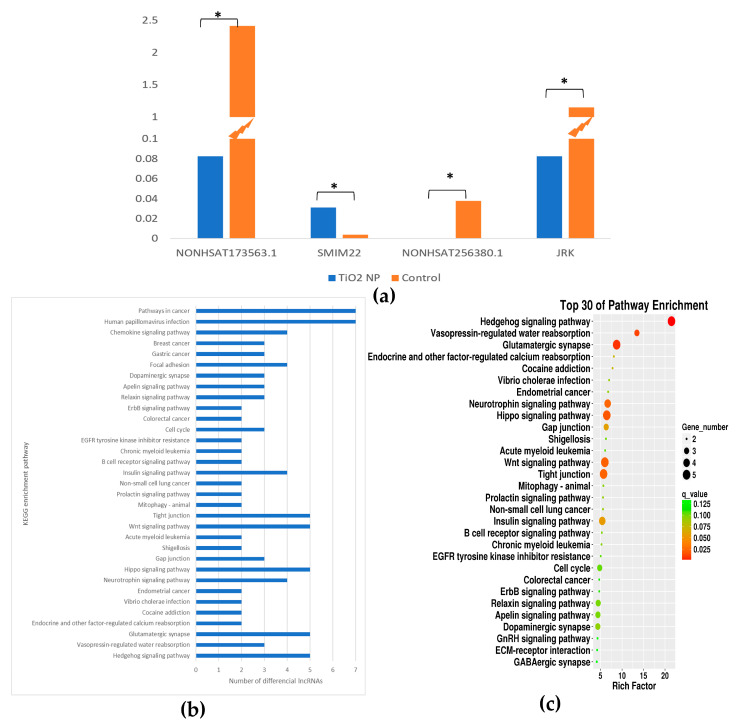
Pathway analysis of TiO_2_ NPs with concentrations of 0 and 100 μg/mL. A bar graph of differential lncRNAs numbers in different pathways of KEGG was drawn (**a**). A KEGG enrichment analysis bubble plot was drawn in the descending order of q value corresponding to each entry in the TiO_2_ NP treatment group (**b**). The relative content of the two lncRNAs and corresponding mRNAs in the TiO_2_ NP treatment and the control group was drawn in a histogram (* *p* < 0.05) (**c**).

The target gene of differential lncRNA was intersected with mRNA. As a result, the lncRNA NONHSAT173563.1 was down-regulated and the matching mRNA SMIM22 was up-regulated (*p* < 0.05). The lncRNA NONHSAT256380.1 was down-regulated and the matching mRNA JRK was down-regulated (*p* < 0.05) (Figure 6a). The changes in the two matching mRNAs were statistically significant.

KEGG enrichment analysis was performed on the intersecting gene. The results showed that the Hedgehog signaling pathway, Vasopressin-regulated water reabsorption, and Glutamatergic synapse were the three most significant pathways of enrichment (*q* < 0.05) (Figure 6b,c). In the Hedgehog signaling pathway, NONHSAT041057.2, NONHSAT091417.2, NONHSAT250525.1, NONHSAT056661.2, and MSTRG.32312.1 changed.

## 4. Discussion

TiO_2_ NPs are exposed to the human body through many pathways and have adverse effects on human health. Moreover, the liver is the target organ of oral exposure to TiO_2_ NPs. The objective of this study was to analyze the effects of TiO_2_ NP exposure on the expression profile of lncRNAs and we tried to understand the potential mechanism of hepatotoxicity through bioinformatics analysis. Through the differential lncRNA analysis and lncRNA–mRNA network, we found that TiO_2_ NPs could induce a change in the expression profile of lncRNAs and may interfere with the Hedgehog signaling pathway and Glutamatergic synapse, eventually leading to hepatotoxicity.

From the CCK-8 assay, TiO_2_ NPs can be slightly toxic to human liver cells. However, many researchers have found that hepatotoxicity is one of the target organ effects of oral exposure to TiO_2_ NPs [17,43]. Geraets et al. [43] investigated the tissue distribution and blood kinetics of various TiO_2_ NPs in rats and found that the liver was identified as the main target tissue, followed by the spleen and lung. Another study found that the liver was the tissue most sensitive to TiO_2_ NP-induced oxidative stress [44]. Many in vivo studies have found that TiO_2_ NPs may produce ROS and promote oxidative stress and liver inflammation [44,45,46]. Sprague-Dawley rats were orally exposed to 0, 2, 10, and 50 mg/kg TiO_2_ NPs for 90 days and were found to induce tissue-specific oxidative stress and elemental imbalance in the liver [44]. In addition, many in vitro studies have found that TiO_2_ NPs can induce damage to hepatocyte line cells [47]. Current major toxicity mechanisms may exert cytotoxic effects on the structure and function of the liver by inducing oxidative stress, inflammation, and apoptosis [16,48,49]. Oxidative stress, considered a common mechanism of the toxicity in NPs, can damage lipids, carbohydrates, proteins, and DNA, ultimately leading to hepatotoxicity [50]. Azim et al. treated mice with anatase TiO_2_ NPs (21 nm, 150 mg/kg/day) for 2 weeks and then added three kinds of antioxidants (idebenone, carnosine, and vitamin E) for 1 month. They finally found that TiO_2_ NPs significantly injured liver function and can be alleviated after the use of antioxidants [49]. This study attempted to further understand the new mechanism of hepatotoxicity from the perspective of epigenetics and found that lncRNAs may play an important role.

In the study, some changes in lncRNAs and changes in the mRNAs matched with differential lncRNAs occurred, with statistical significance, implying that epigenetics may play a role in hepatotoxicity. Epigenetics is an important link in the regulation of genotype and phenotype. The regulation and dysregulation of genotype and phenotype often lead to the occurrence of diseases and have long-term negative effects. According to the 3R principle, epigenetics is also gradually being used in the toxicity study of nanomaterials. Some studies have also found that, in addition to genetic and cytotoxic effects, they can also affect the epigenome of target cells [23,51]. Lu et al. exposed human and murine macrophages (THP-1 and RAW264.7, respectively) and human small-airway epithelial cells (SAECs) to environmentally relevant concentrations of TiO_2_ NPs, resulting in modest alterations in DNA methylation [51]. Another study also found that low concentrations of TiO_2_ NPs can alter the enzymes responsible for epigenetic modifications [52]. Because their concentrations are well below sublethal levels, changes in DNA methylation can serve as good biomarkers of early exposure to TiO_2_ NPs. Therefore, epigenetic studies are critical for a complete assessment of potential risks from nanoparticle exposure.

In recent years, lncRNAs have become an important class of regulators of gene expression and epigenetic regulation [53]. Some reports found that lncRNAs play a role in cell-cycle regulation, apoptosis, and the establishment of cellular identity [54,55]. Changes in the expression of lncRNAs have been proven to be linked with cancer (e.g., prostate cancer) and several neurological disorders [31,56]. One study proposed that the use of electrochemical nucleic acid sensors is very sensitive to lncRNA HULC detection, providing a new alternative for clinical HCC diagnosis [57]. The study did find that certain lncRNAs (such as NONHSAT256380.1 and NONHSAT173563.1) showed remarkable changes, which may be prevalent to the hepatotoxicity of TiO_2_ NPs. Therefore, lncRNAs can help to study the mechanism of hepatotoxicity in more depth and explore the role of epigenetic regulation in hepatotoxicity.

In addition, small integral membrane protein 22 (SMIM22, CASIMO1), matched with the up-regulated lncRNA (NONHSAT173563.1), has been shown to play a key role in carcinogenesis, cell proliferation, and cell lipid homeostasis [58]. The depletion of Jrk helix-turn-helix protein (JRK, JH8, jerky), matched with the down-regulated lncRNA (NONHSAT256380.1), inhibits the transcriptional activity of β-catenin and reduces cell proliferation, and it has been validated for carcinogenic effects in primary tumors [59]. From the result of KEGG enrichment analysis, TiO_2_ NPs could interfere with the Hedgehog signaling pathway, which played a key role in tissue development and dryness. The imbalance in the Hedgehog signaling pathway was present in many different tumors, such as skin, brain, liver, and gallbladder [60]. There are three homology genes for Hedgehogs in mammals: Sonic Hedgehog (SHH), Indian Hedgehog (IHH), and Desert Hedgehog (DHH) [61]. Hedgehog signaling is controlled by two receptors, Patched (Ptc) and Smoothened (Smo), on the membrane of the target cell [62]. These unique signaling molecules are highly expressed in most malignant tissues and have been considered biomarkers for progression and prognosis [63,64]. Additionally, many in vitro studies have found that chronic liver damage or liver cancer may activate the sonic hedgehog (SHH) pathway [65,66].

The main advantage of this article is the use of epigenetics to study the alterations in the lncRNA expression profile induced by TiO_2_ NPs in hepatotoxicity. In the future, the influence of oral exposure to nano-titanium dioxide on epigenetics and related mechanisms can be further studied. However, this study also has some drawbacks. Firstly, this study lacks more in-depth studies on screened lncRNAs and, secondly, the verification of this study is at the mRNA level, so there is a lack of PCR verification at the lncRNA level. Therefore, we will next conduct more in-depth studies on differential lncRNAs, such as knocking out relevant genes to study their impact on subsequent functions. We will also further focus on the effects of apoptosis or genetic damage of TiO_2_ NPs.

## 5. Conclusions

The present study focused on alterations in the lncRNA expression profile in HepG2 cells after exposure to TiO_2_ NPs and its potential role in the mechanism of hepatotoxicity. It was demonstrated that exposure to TiO_2_ NPs could induce a series of differential lncRNAs, represented by lncRNA NONHSAT256380.1 and lncRNA NONHSAT173563.1. Meanwhile, the target gene analysis indicated that these differential lncRNAs may be involved in hepatotoxicity by interfering with the Hedgehog signaling pathway. Two lncRNA–mRNA networks, including lncRNA NONHSAT256380.1-JRK and lncRNA NONHSAT173563.1-SMIM22, were verified. It was suggested that epigenetics may play an important role in the mechanism of hepatotoxicity induced by TiO_2_ NPs.

## Data Availability

Not applicable.
